# A Recombinant Subviral Particle-Based Vaccine Protects Magpie (*Pica pica*) Against West Nile Virus Infection

**DOI:** 10.3389/fmicb.2019.01133

**Published:** 2019-06-05

**Authors:** Nereida Jiménez de Oya, Estela Escribano-Romero, María-Cruz Camacho, Ana-Belén Blazquez, Miguel A. Martín-Acebes, Ursula Höfle, Juan-Carlos Saiz

**Affiliations:** ^1^ Departamento de Biotecnología, Instituto Nacional de Investigación y Tecnología Agraria y Alimentaria (INIA), Madrid, Spain; ^2^ Grupo de Sanidad y Biotecnología SaBio, Instituto de Investigación en Recursos Cinegéticos IREC, CSIC-UCLM-JCCM, Ciudad Real, Spain

**Keywords:** flavivirus, West Nile virus, birds, vaccines, transmission, protection, herd immunity

## Abstract

The mosquito-borne West Nile virus (WNV) is a highly neurovirulent *Flavivirus* currently representing an emergent zoonotic concern. WNV cycles in nature between mosquito vectors and birds that act as amplifier hosts and play an essential role in virus ecology, being, thus, WNV a threat to many species. Availability of an efficient avian vaccine would benefit certain avian populations, both birds grown for hunting and restocking activities, as well as endangered species in captive breeding projects, wildlife reservations, and recreation installations, and would be useful to prevent and contain outbreaks. Avian vaccination would be also of interest to limit WNV spillover to humans or horses from susceptible bird species that live in urbanized landscapes, like magpies. Herein, we have addressed the efficacy of a single dose of a WNV recombinant subviral particle (RSP) vaccine in susceptible magpie (*Pica pica*). The protective capacity of the RSP-based vaccine was demonstrated upon challenge of magpies with 5 × 10^3^ plaque forming units of a neurovirulent WNV strain. A significant improvement in survival rates of immunized birds was recorded when compared to vehicle-inoculated animals (71.4 vs. 22.2%, respectively). Viremia, which is directly related to the capacity of a host to be competent for virus transmission, was reduced in vaccinated animals, as was the presence of infectious virus in feather follicles. Bird-to-bird transmission was recorded in three of six unchallenged (contact) magpies housed with non-vaccinated WNV-infected birds, but not in contact animals housed with vaccinated WNV-infected magpies. These results demonstrate the protective efficacy of the RSP-based vaccine in susceptible birds against WNV infection and its value in controlling the spread of the virus.

## Introduction

Human and animal health has to face changes in the ecology of pathogens resulting from globalization and climate warming. Flaviviruses represent one of these emerging challenges and are currently spreading worldwide, as exemplified by the recent pandemic of Zika virus (Saiz et al., 2016) and the increasing outbreaks of WNV (Martin-Acebes and Saiz, 2012; Munoz et al., 2018). Therefore, development of efficient vaccines to control them is an urgent need.

WNV is a highly neurovirulent pathogen naturally maintained in an enzootic cycle between ornithophilic mosquitoes and certain birds. It is responsible for sporadic outbreaks in humans and horses, in which the infection is mainly asymptomatic even though it can also have a fatal outcome and result in epidemics and epizootics (Martin-Acebes and Saiz, 2012). Hundreds of bird species are susceptible to the infection, and several develop competent viremia to efficiently transmit the virus to vectors, thus playing an essential role in virus maintenance (Komar et al., 2003). WNV-associated mortality has been described in domestic (Swayne et al., 2001; Wunschmann and Ziegler, 2006; Eckstrand et al., 2015) and wild birds (Ludwig et al., 2002; Ward et al., 2006; LaDeau et al., 2007), including some adapted to human environments (Foss et al., 2015), and in endangered species (Yaremych et al., 2004; Jimenez-Clavero et al., 2008). To date, WNV licensed vaccines are only available for use in equids. Therefore, assessment of the protective capability of vaccine candidates in birds that are natural hosts and virus amplifiers can be very useful to control WNV outbreaks.

Herein, we have assayed the effectiveness of a WNV-recombinant subviral particle (RSP)-based vaccine in the magpie (*Pica pica*), a member of the family Corvidae, whose habitats include cultivated land and suburban areas, and that is highly susceptible to WNV and possibly a transmission competent species (Jimenez de Oya et al., 2018). The RSPs, also referred to as virus-like particles (VLPs), result from the co-expression of the prM and E glycoproteins of WNV and mimics immunogenic properties of the whole virion. RSPs have already shown their potential as vaccine candidates for WNV (Merino-Ramos et al., 2014), tick-borne virus encephalitis (Heinz et al., 1995), Japanese encephalitis virus (Hunt et al., 2001), and Murray Valley encephalitis virus (Kroeger and McMinn, 2002) in the mouse model. Here, we show that immunization with a single dose of the WNV-RSP-based vaccine protects magpies upon challenge with a neurovirulent strain of WNV. Moreover, the vaccine significantly reduced viremic titres below the threshold to consider a host as a competent amplifier and significantly reduced viral burden in feathers, remarking its value as a useful tool to control WNV.

## Materials and Methods

### Ethics Statement

Animals were handled according to the guidelines of the European Community 2010/63/UE. Magpies were captured between April and June 2018 in different hunting locations in South-Central Spain (permit 346760, Regional Government of the Autonomic Community of Castilla-La Mancha, Spain) and housed as previously described (Jimenez de Oya et al., 2018). All protocols involving animals were approved by the Committee on Ethics of animal experimentation of the host Institution (INIA’s permit number 2018-004). Infectious virus manipulation was carried out in dedicated BSL-3 facilities in strict accordance to biosecurity rules. Magpies were provided with food and water *ad libitum* and were monitored daily through the duration of the experiment. Birds that met the criteria established for humanitarian endpoint (e.g., severe signs of WNV neuropathology, excessive weight loss, acute deviation of behavior, etc.), as well as all surviving ones at the end of the experiment, were euthanized with sodium pentobarbital (Dolethal, Vetoquinol, Madrid, Spain).

### Recombinant Subviral Particles (RSPs)

RSPs were purified from the supernatant of a WNV-HeLa3 cell line that secretes them constitutively (Merino-Ramos et al., 2014) by centrifugation through a sucrose gradient as described (Martin-Acebes et al., 2014). Immunodot analyses were performed to select peak fractions containing WNV-specific E protein (Merino-Ramos et al., 2014), the amount of which was estimated by Bradford assay using a standard curve of bovine serum albumin.

### Bird Vaccination, Challenge, and Sampling

**Figure 1:**
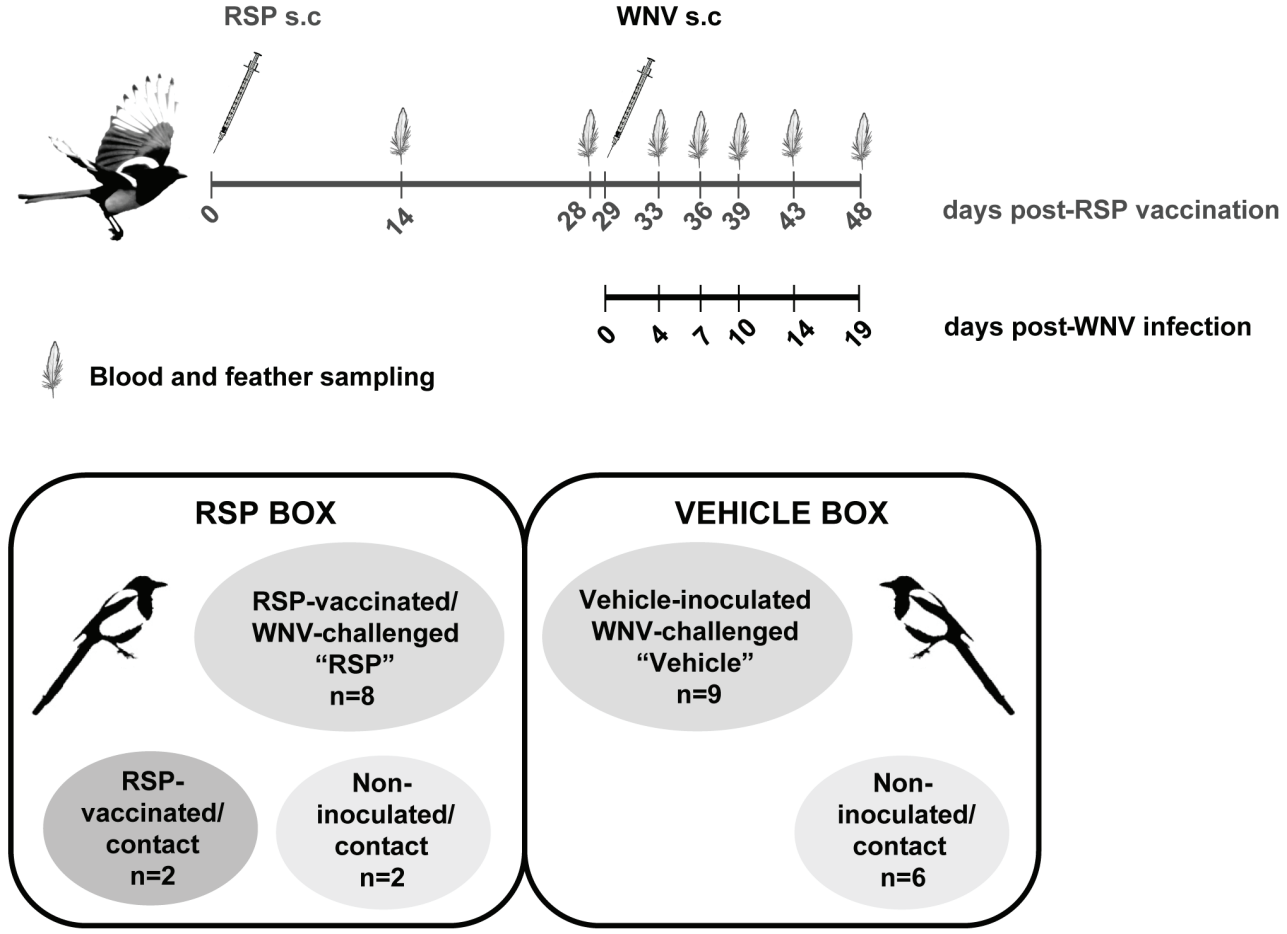
Experimental design. Schematic representation of the immunization, sampling schedule, and the distribution of the magpies in the experimental boxes.

Birds were aged based on plumage and molt patterns, sampled, and tested for the presence of WNV neutralizing antibodies (NAbs) in their sera by plaque reduction neutralization test (PRNT) as described (Vazquez-Calvo et al., 2017). Real time RT-PCR was used to test the presence of WNV genome in serum and feather follicles, as described previously (Jimenez de Oya et al., 2018). A final group of 27 juvenile (less than 1 year old) magpies negative for WNV NAbs and WNV genome was transported to our biosafety level 3 (BSL-3) facilities where they were housed in 2 appropriately equipped separate cages, 12 in the RSP-box, and 15 in the vehicle-box, as described (Figure 1; Jimenez de Oya et al., 2018). After 1 week of adaptation, animals were weighed and bled *via* the jugular vein for pre-inoculation serology.

A group of 10 magpies was immunized once subcutaneously in the thigh with 50 μg/magpie of purified RSPs administered (1:1) with aluminum hydroxide gel adjuvant (Alhydrogel®, InvivoGen, San Diego, USA), while another group (vehicle) of 9 were inoculated with adjuvant alone by the same route. Twenty-nine days later, magpies (eight RSPs-immunized and nine vehicle-inoculated) were subcutaneously challenged in the inguinal space (*crural patagium*) with 5 × 10^3^ plaque forming units (pfu)/animal of WNV strain NY-99 (GenBank accession no. KC407666, Merino-Ramos et al., 2014) diluted in 200 μl of Eagle Minimum Essential Medium (EMEM, BioWhittaker, Lonza, Verviers, Belgium). The inoculum was back-titrated to confirm the inoculated dose. Ten uninfected birds (six in the vehicle-inoculated group and four in the RSPs-vaccinated group, two immunized, and two not) were housed with the infected magpies, as control for bird-to-bird virus transmission (Figure 1).

Birds were bled from the jugular vein before vaccination, at days 14 and 28 post-vaccination, and at 4, 7, 10, 14, and 19 days post-infection (d.p.i.), corresponding to days 33, 36, 39, 43, and 48 post-vaccination. At the indicated times post-infection, birds were weighed and at least two of any growing feathers containing pulp were collected (contour feathers of the body or head, or tail or wing coverts, depending on the individual). The number of feathers employed for pulp extraction was adapted according to their size to work with approximately equal amounts of pulp (Figure 1).

### Serological and Virological Assays

Whole blood (0.5 ml/bird) was allowed to coagulate at 4°C overnight and the serum was collected after centrifugation at 1,300× g at 4°C for 10 min, and kept at −80°C until use. Serum was diluted 1/10 and filtered through 0.2 μm pore size filters (Acrodisc® syringe filters, Pall Corporation, Port Washington, New York, US). Serial serum dilutions were titrated by plaque assay as described (Martin-Acebes et al., 2013).

The pulp of feather follicles was extracted from the *calamus* of growing feathers with fine point tweezers, covered with 0.5 ml of fresh Eagle minimum essential medium (EMEM) supplemented with glutamine and penicillin-streptomycin, and stored at −80°C until use. Detection of infectious virus in the follicles was performed, after one cycle of quick freeze and thaw, in serial dilutions as above, starting from a filtered 1/10 dilution.

Detection of WNV NAbs in sera was performed by plaque reduction neutralization test (PRNT) using twofold dilutions of previously filtered, heat inactivated (30 min at 56°C) sera (Vazquez-Calvo et al., 2017). Antibody titres were established by calculating the inverse of the highest dilution (minimum dilution 1:20) capable of inhibiting virus replication by 90% (PRNT_90_).

### Statistical Analyses

Data analysis was performed using Graph Pad Prism 6 (Graph Pad Software, Inc., San Diego, CA, 2005). Kaplan-Meier survival curves were analyzed by a log-rank (Mantel Cox) test. The analysis of variance (ANOVA) with Bonferroni’s correction was applied for multiple comparisons. For single comparison and non-parametric data, U Mann-Whitney tests were performed. Significant differences are shown in the figures represented by asterisks *p < 0.05, **p < 0.01, ***p < 0.001, and ****p < 0.0001.

## Results

### Vaccine Safety

None of the vaccinated animals showed evidence of adverse reaction after vaccine administration, but most of them developed a thick walled fibrous cyst filled with whitish material of 2–8 mm in diameter at the site of inoculation.

### Protective Role of the Vaccine

**Figure 2:**
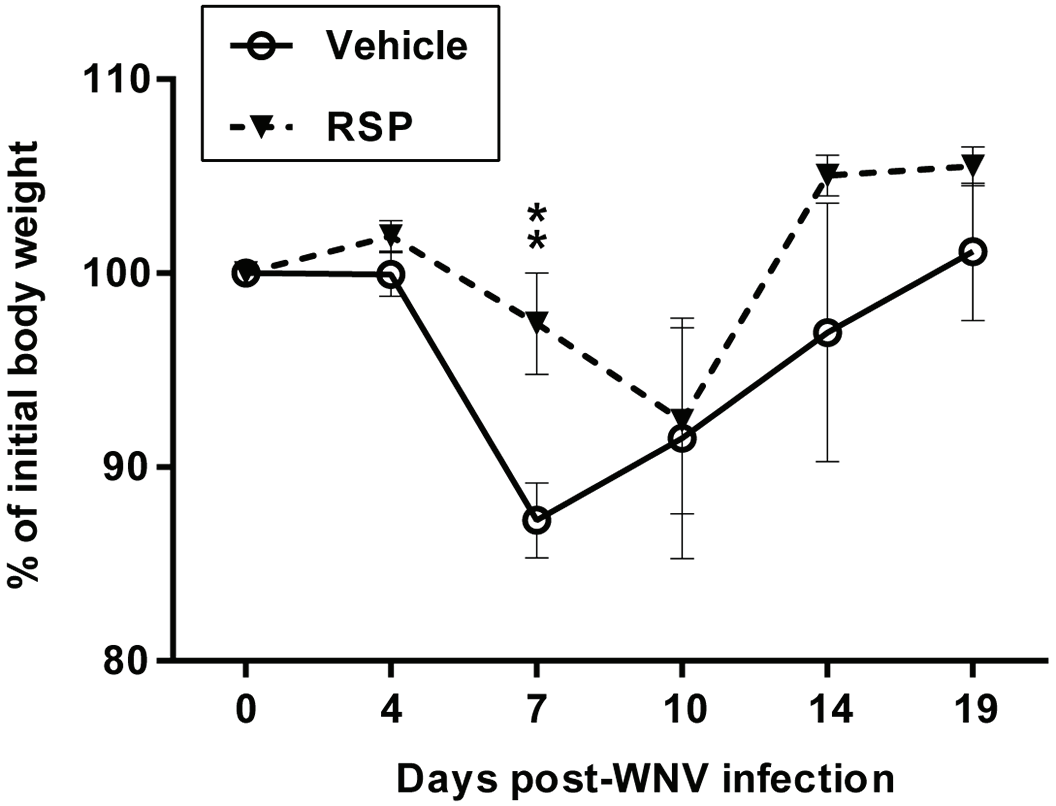
Time-course of body weight changes of magpies challenged with WNV. Body weight measurements of non-vaccinated (vehicle) and vaccinated (RSP) magpies after WNV challenge expressed as the percentage of the initial body weight. Data are presented as mean ± SEM. Statistically significant differences are represented by asterisks (**p < 0.01).

**Figure 3:**
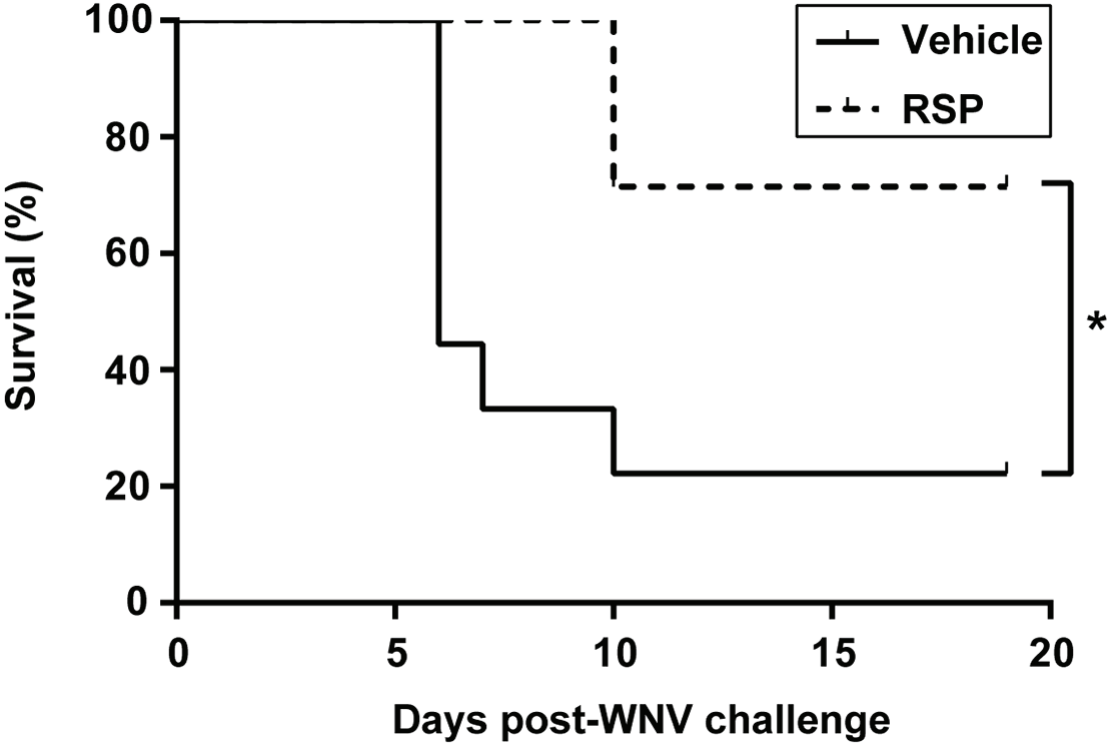
WNV-RSP-based vaccine increases survival rates in magpies challenged with WNV. Survival rates of non-vaccinated (vehicle) and vaccinated (RSP) magpies after WNV challenge. Statistically significant differences are represented by an asterisk (*p < 0.05).

Both vehicle-inoculated (*n* = 9) and vaccinated (*n* = 8) groups were challenged with 5 × 10^3^ pfu/magpie of the neurovirulent WNV-NY99 strain 29 days post-immunization (Figure 1). Magpies that succumbed to the infection showed disease-related signs (less than 12 h prior to death) such as ruffled feathers, lethargy, loss of equilibrium, and ataxia, as did sick animals before being euthanized according to endpoint criteria. Weight loss was observed in both infected groups of birds at days 7 and 10 p.i., although in vaccinated birds, it was significantly less pronounced and delayed in comparison to unvaccinated magpies (Figure 2). From there on, surviving animals began to gain weight until the end of the experiment. Survival rate in vehicle-inoculated birds (22.2%, 2/9) was significantly lower than in vaccinated magpies (71.4%, 5/7) (Figure 3). One vaccinated bird was sacrificed at 7 d.p.i. for the purpose of histopathologic analysis and, thus, it was excluded from the survival analysis. Mortality kinetics in vaccinated magpies (dead at day 10 p.i.) was delayed as compared to that of non-immunized birds, which died between days 6–10 p.i. (median survival time, MST, 6 d.p.i.).

### Humoral Response

**Figure 4:**
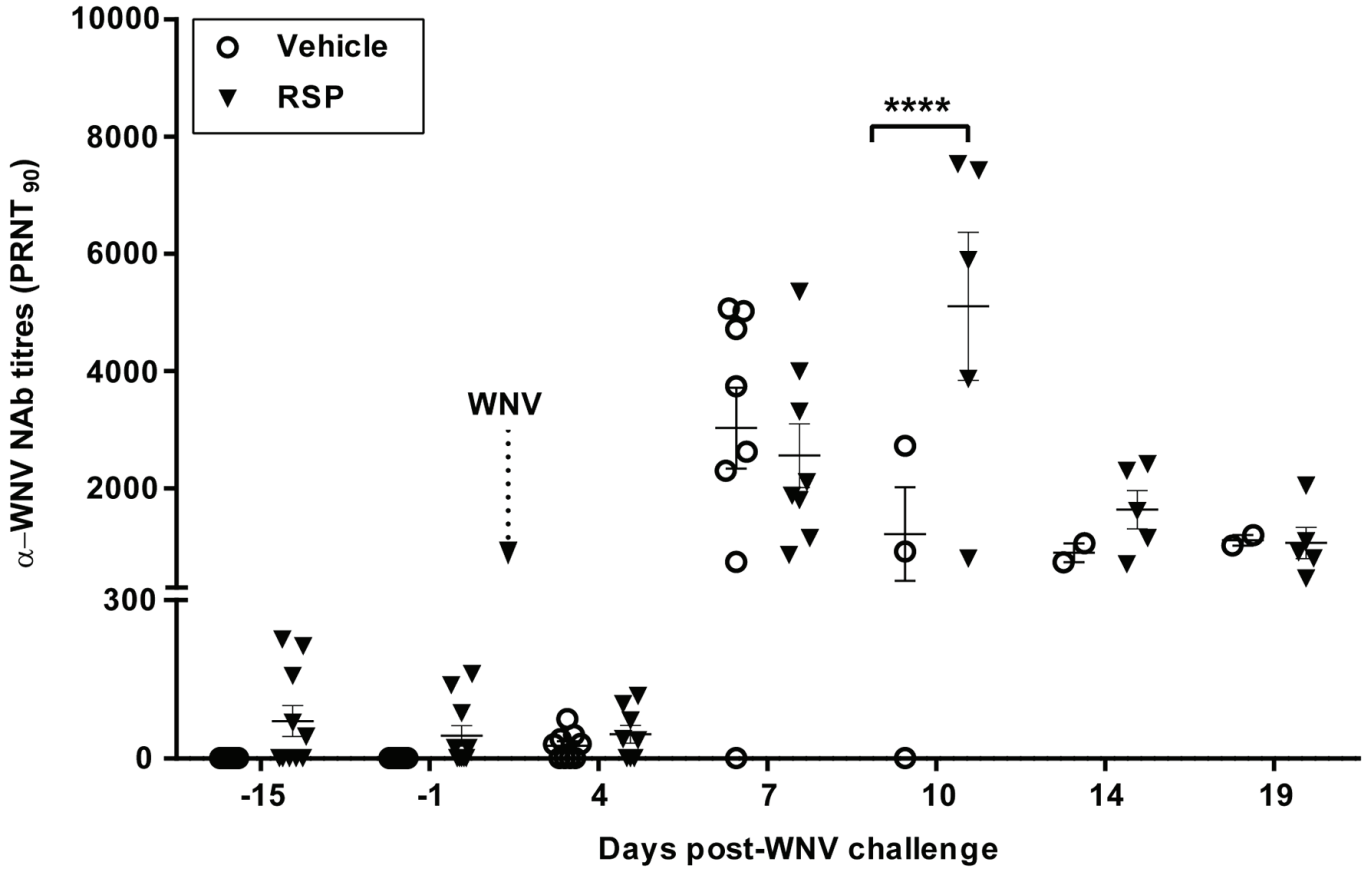
Humoral immune response. Neutralizing antibody (NAb) titres in the sera of non-vaccinated (vehicle) and vaccinated (RSP) magpies measured by plaque reduction neutralization test (PRNT_90_) at different time points previous to and after WNV challenge. Data are presented as mean ± SEM. Statistically significant differences are represented by asterisks (****p < 0.0001).

None of the birds presented anti-WNV NAbs, measured as PRNT_90_, nor WNV genome prior to immunization. The RSP-based vaccine elicited detectable WNV NAbs in five vaccinated birds at day 14 after immunization (Figure 4). WNV challenge induced humoral immunity in both vaccinated and non-vaccinated groups, although with different kinetics and magnitude, peaking at 7 and 10 d.p.i. in vehicle-inoculated and RSPs-vaccinated magpies, respectively, with significantly higher antibody titres in vaccinated than in vehicle-inoculated magpies at 10 d.p.i.

### Control of Viral Infection

**Figure 5:**
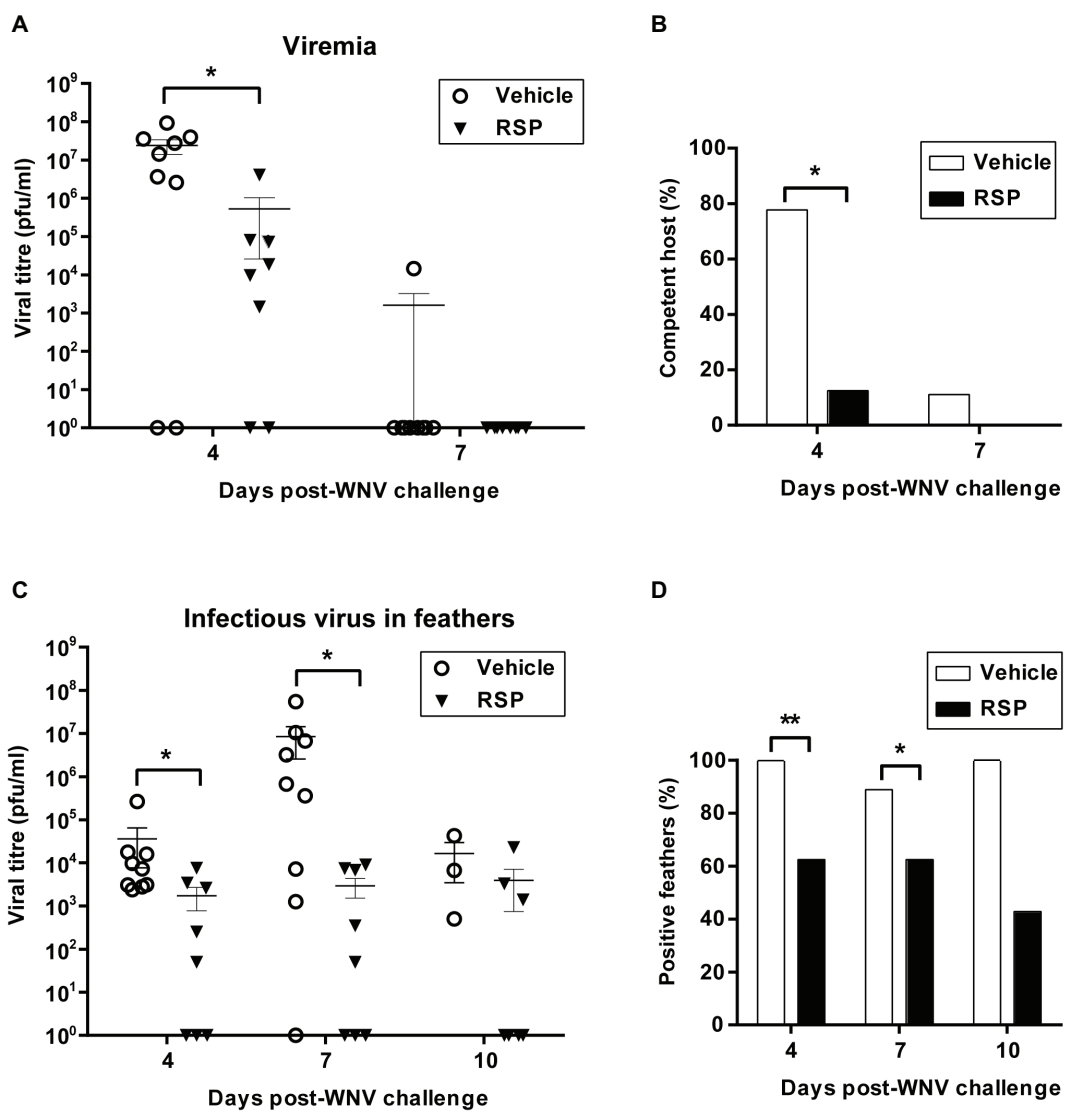
The WNV-RSP-based vaccine reduces viremia and infectious virus in feather follicles of magpies infected with WNV. Viremia titres **(A)**, percentages of hosts that are competent (i.e., birds with viremic titres above 10^5^ pfu/ml) **(B)**, viral titres in feather follicles **(C)**, and percentages of animals with virus in feather follicles **(D)** in non-vaccinated (vehicle) and vaccinated (RSP) magpies after WNV challenge. Data are presented as mean ± SEM. Statistically significant differences are represented by asterisks (*p < 0.05 and **p < 0.01).

Viremia found in vaccinated magpies was significantly lower at 4 d.p.i. when compared with non-primed birds, and at day 7 p.i., only one vehicle-inoculated bird presented measurable viremia (Figure 5A). A titre above 10^5^ pfu/ml has been established to consider a viremic bird as a competent host to transmit the virus to most WNV competent mosquito vectors (Komar et al., 2003). At 4 d.p.i., the number of vaccinated magpies with viremia above this competence threshold was significantly lower than in the vehicle-inoculated group (12.5 vs. 77.8%, respectively) (Figure 5B).

Similarly, viral titres found in feather follicles were significantly reduced in vaccinated magpies in comparison to non-vaccinated birds at 4 and 7 d.p.i. (Figure 5C). Moreover, the proportion of animals with infectious virus in follicles was consistently lower in vaccinated than in non-vaccinated magpies at the different time points analyzed (Figure 5D).

### Virus Horizontal Transmission

**Figure 6:**
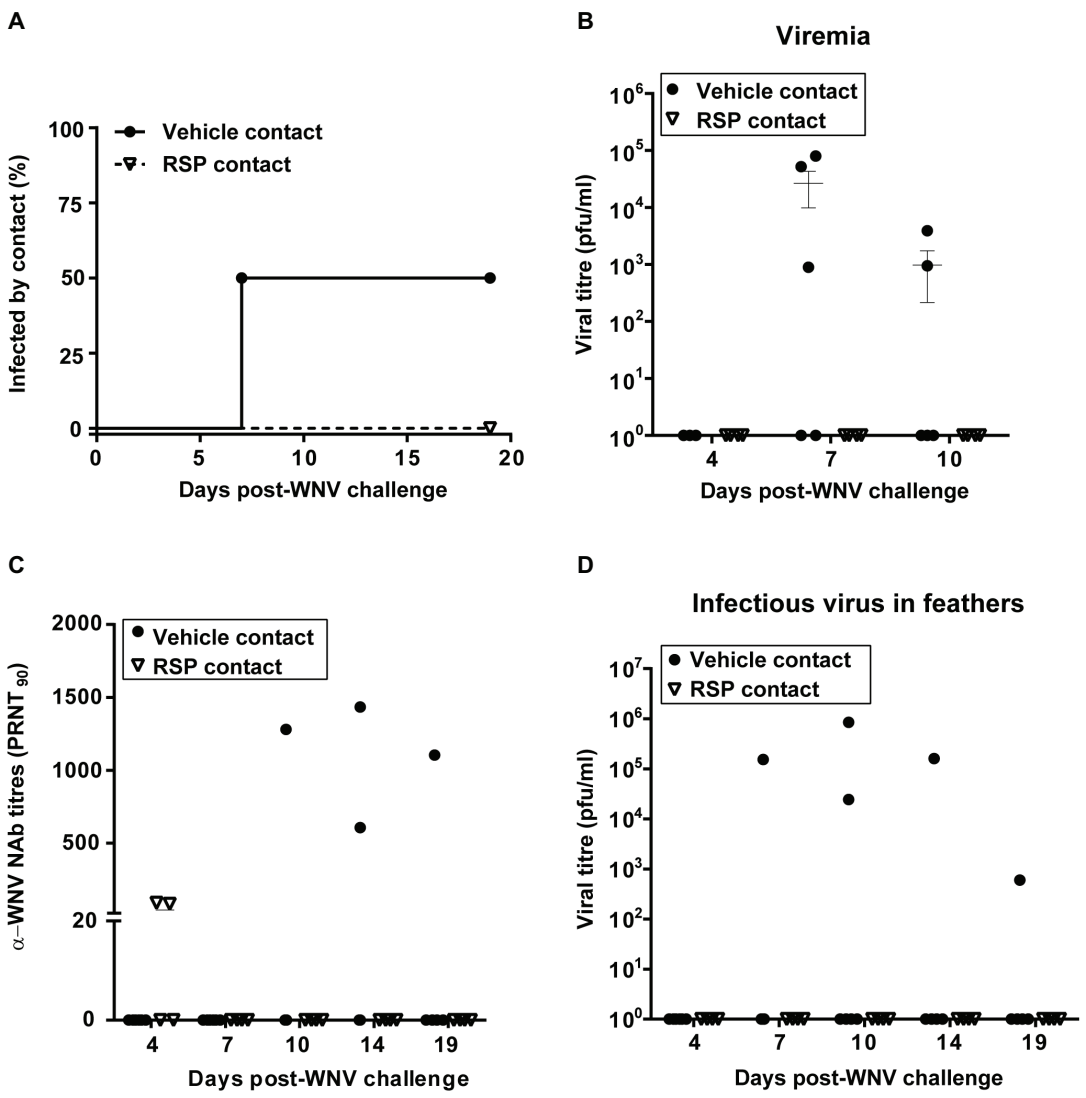
Bird-to-bird WNV transmission occurs only in non-vaccinated magpies. Percentage of animals infected by bird-to-bird contact **(A)**, viremia titres **(B)**, neutralizing antibody (NAb) titres, PRNT_90_ **(C)**, and viral titres in feather follicles **(D)** in contact-infected magpies. Solid and empty symbols represent contact birds housed with non-vaccinated and vaccinated animals, respectively.

Contact animals that were not challenged were housed with WNV-challenged birds in both groups (four in the RSPs-vaccinated and six in the vehicle-inoculated group) throughout the experiment to explore bird-to-bird virus transmission. In the vaccinated group, two of the contact magpies had been vaccinated and two were not (Figure 1). Bird-to-bird contact was analyzed based on presence of NAbs and infectious virus in sera and feather follicles. Remarkably, viral transmission was observed in contact birds housed with vehicle-inoculated birds, but not in those allocated with immunized cage-mates (Figure 6A), which indicates the capability of the vaccine to confer herd immunity. Bird-to-bird viral transmission was confirmed by detection of viremia in 3/6 (50%) contact magpies housed with vehicle-inoculated cage-mates at 7 d.p.i. (Figures 6A,B) and by the death of one of them. Viremia was detected in these contact birds 3 days after the peak of viremia of their challenged cage-mates (Figure 6B). Contact-infected magpies presented NAbs from day 10 p.i., again with some delay in comparison to the experimentally challenged birds (Figure 6C). Infectious virus was also found in the feather follicles of contact-infected birds from 7 to 19 d.p.i., depending on the animal (Figure 6D), but also with a delay in comparison to their challenged cage-mates. These results support that the control of the virus after WNV-RSPs vaccination averts bird-to-bird infection, as none of the contact magpies included in the vaccinated group was infected.

## Discussion

There is a current (re)-emergence of arthropod-borne viruses, such as West Nile, dengue, or Zika virus, due to the arrival and establishment of their vectors in new geographical regions, global trade, and urbanization. Accordingly, a greater number of autochthonous cases are being reported in non-endemic areas, as evidenced by the recent pandemic of Zika virus in America (Saiz et al., 2016) or dengue cases in European countries.^1^

WNV has a worldwide distribution and had been associated mainly with rare and infrequent epidemics/epizootics. However, the virus re-emerged in the 90s, being responsible of more frequent and severe outbreaks in humans, horses, and birds, as exemplified by the outbreak in 1999 in New York (Martin-Acebes and Saiz, 2012). Since then, several outbreaks have been described around the world. In fact, the number of WNV human cases in Europe has worryingly increased during 2018 to up to 1,875 confirmed ones, with 115 deaths,^2^ locally concurrent with wild bird mortality, namely, in magpies. Therefore, there is a clear need for the implementation of more efficient surveillance and control programs to combat WNV.

Nowadays, the only approved vaccines are for use in equids. These vaccines have demonstrated to be highly efficient in drastically reducing infectious rates among horses in the US (Martin-Acebes and Saiz, 2012; Iyer and Kousoulas, 2013). The availability of vaccines for use in birds, the natural hosts of the virus, which can break the viral cycle by helping to control it, will be highly useful, mainly during outbreaks. Such vaccines could be used in birds held in captivity in zoos, recreational installations, wildlife rehabilitation, and endangered species breeding centers, in surveillance programs, and even in birds grown for restocking or hunting activities that are yearly released by the thousands into the environment in many countries. In fact, DNA vaccines, either experimental or commercially available for equine use, have been previously tested in fish crows (Turell et al., 2003), American crows (Bunning et al., 2007) scrub jays (Wheeler et al., 2011), red-tailed hawks (Redig et al., 2011), and falcons (Fischer et al., 2015). Likewise, recombinant vaccines were evaluated in geese (Jarvi et al., 2008), red-legged partridges (Escribano-Romero et al., 2013), and falcons (Angenvoort et al., 2014), as were inactivated ones in falcons (Angenvoort et al., 2014) and geese (Malkinson et al., 2001). This latter one was even administered to 1,800 geese in a follow-up study with a survival rate over 96% (Samina et al., 2005). Even more, a prospective vaccination of condors in California before the virus was introduced there was claimed to act as a potential barrier from subsequent WNV infections (Chang et al., 2007). In most of these studies, birds were challenged 6–10 weeks after immunization, and two or three doses were necessary to observe significant antibodies titres, reduction of viremia levels and, when analyzed, of viral shedding. However, in none of these reports, the induction of herd immunity was evaluated.

To date, more than 300 wild and domestic avian species have been described as susceptible to WNV infection (Komar et al., 2003), being corvids highly prone to it and important virus amplifiers (Komar et al., 2003; Lim et al., 2015; Jimenez de Oya et al., 2018) thus playing a key role in the epidemiology of the virus (Eidson, 2001; Eidson et al., 2001a,b; Reisen et al., 2006; Hinton et al., 2015). In fact, WNV epidemics in the US were associated with high crow mortality and have even led to a significant decrease of native crow species (Reisen et al., 2006; LaDeau et al., 2007; Ernest et al., 2010; Foss et al., 2015). Corvids seem to be also involved in the WNV endemic cycle in human habitats in Europe (Calistri et al., 2010). Among them, the magpie is one of the most abundant corvids in Europe (Madge, 2018). Magpies live in urbanized landscapes and constitute a feeding preference of *Culex pipiens*, a main WNV-vector to humans (Rizzoli et al., 2015). In addition, it has been reported to be highly susceptible to viral infection and a possible source of WNV transmission (Jimenez de Oya et al., 2018), making of this bird species an interesting player in WNV ecoepidemiology. Similar to American Yellow-billed magpies (*Pica nutalli*) and American crows (*Corvus brachyrhynchos*), Eurasian magpies form large communal roosts outside the breeding season that could be important for WNV persistence during winter by bird-to-bird transmission, as has been suggested for American crows (Hinton et al., 2015).

In the present study, we have addressed the capability of a RSPs vaccine to protect magpies against a lethal dose of WNV NY-99 strain, which is highly virulent in American crows (Brault et al., 2004, 2007), and whose virulence and pathogenicity in animal models, both mice and magpies (Merino-Ramos et al., 2014; Jimenez de Oya et al., 2018), are similar to those of currently circulating lineage 2 WNV strains in Europe (Petrovic et al., 2013).

RSPs are potent inducers of both B cell responses (Zhang et al., 2009; Tan and Jiang, 2014), essential to control WNV-infection (Diamond et al., 2003), and T cell responses (Tan and Jiang, 2014; Pitoiset et al., 2015), important in viral clearance (McMurtrey et al., 2008), being consequently an excellent choice for vaccine development. In this sense, we have previously reported that the RSP vaccine candidate used here protects mice experimentally infected with WNV strains of different lineages (Merino-Ramos et al., 2014). Now we have confirmed the protective capability of the vaccine candidate in a virus natural host, as the survival rate (71.4%) of the vaccinated magpies was significantly higher than that of non-vaccinated birds (22.2%), in which the percentage of survival was similar (30%) to that previously reported in experimentally infected naïve magpies (Jimenez de Oya et al., 2018).

By day 14 after immunization, NAbs were detected in 50% of the animals (5/10, mean PRNT_90_ = 70.6 ± 92.9) upon a single dose of the vaccine. By day seven after viral infection, and with the exception of one vehicle-inoculated magpie, NAbs titres were detected in all challenged birds, but titres were higher in non-vaccinated animals than in vaccinated ones (mean PRNT_90_ = 3,027 ± 1,949 vs. PRNT_90_ = 2,555 ± 1,539, respectively), probably due to a more prominent and generalized viral replication that enhances the humoral immune response (Honke et al., 2011). Nevertheless, at day 10 p.i. NAb titres were significantly higher in the vaccinated group in comparison to non-vaccinated ones (mean PRNT_90_ = 5,103 ± 2,827 vs. PRNT_90_ = 1,214 ± 1,387, respectively). These results point out that, besides the contribution of the NAbs, the cellular immune response is likely to play a pivotal role in containing infection, an aspect that merits further investigation.

A single dose of vaccine not only partially protected magpies from a lethal WNV infection, but also significantly reduced viremia. Viremia levels were measured at 4 and 7 d.p.i. Only one out of eight (12.5%) vaccinated birds had viremia titres above 10^5^ pfu/ml, high enough to consider an infected bird as a competent host to transmit the virus to the vector (Komar et al., 2003; Turell et al., 2003), while seven out of nine (77.7%) vehicle-inoculated birds reached that threshold. One vehicle-inoculated magpie was still viremic at 7 d.p.i. Similarly, detection of virus in follicles of growing feathers, which could also be considered a source of virus transmission (Banet-Noach et al., 2003; Docherty et al., 2004; Jimenez de Oya et al., 2018), showed that few of the vaccinated magpies were positive, with significantly lower titres, when compared to non-vaccinated ones. In fact, none of the four contact birds housed with vaccinated animals got infected, while three of the six housed with non-vaccinated magpies were viremic, presented infectious virus in feathers, developed neutralizing antibodies, and one of them succumbed to the infection. Therefore, vaccination of the magpies seems to induce herd immunity, diminishing the risk of both host-to-vector and bird-to-bird virus transmission. This could be of importance for the control of WNV persistence if herd immunity is induced in communal roosting species such as magpies and crows, in which bird-to-bird transmission may represent an important mode of viral persistence outside the seasons with vector activity (Hinton et al., 2015; Montecino-Latorre and Barker, 2018), Therefore, bird vaccination would really impact on virus maintenance especially if effective ways of administration, as oral delivery, are developed.

In brief, we herein report the efficacy of a single dose of an anti-WNV RSP-based candidate vaccine in conferring protection to the magpie, a natural virus amplifier host with a key role in WNV ecology. The vaccine has demonstrated to be safe for birds and its production does not require BSL-3 facilities, as RSPs are not infectious, which reduces the cost of production and facilitates its manipulation. It is also worth noting that this type of vaccine could be used without interfering with ongoing WNV surveillance programs since it could enable the differentiation between naturally infected and vaccinated animals (DIVA vaccine). In conclusion, a single dose of the RSPs vaccine protects magpies from WNV, eliciting a neutralizing immune response and interfering with the virus cycle by reducing viremia levels and the risk of horizontal contact.

## Data Availability

The raw data supporting the conclusions of this manuscript will be made available by the authors, without undue reservation, to any qualified researcher.

## Ethics Statement

Animals were handled according to the guidelines of the European Community 2010/63/UE. Magpies were captured between April and June 2018 in different hunting locations in South-Central Spain (permit 346760, Regional Government of the Autonomic Community of Castilla-La Mancha, Spain) and housed as previously described (Jimenez de Oya et al., 2018). All protocols involving animals were approved by the Committee on Ethics of animal experimentation of the host Institution (INIA’s permit number 2018-004). Infectious virus manipulation was carried out in dedicated BSL-3 facilities in strict accordance to biosecurity rules. Magpies were provided with food and water *ad libitum* and were monitored daily through the duration of the experiment. Birds that met the criteria established for humanitarian endpoint (e.g., severe signs of WNV neuropathology, excessive weight loss, acute deviation of behavior, etc.), as well as all surviving ones at the end of the experiment, were euthanized with sodium pentobarbital (Dolethal, Vetoquinol, Madrid, Spain).

## Author Contributions

EE-R, UH, and J-CS contributed in conceptualization. NJO, EE-R, M-CC, A-BB, MM-A, UH, and J-CS contributed in methodology and investigation. A-BB, MM-A, NJO, EE-R, and J-CS contributed in formal analysis. NJO and J-CS contributed in writing original draft. NJO, EE-R, M-CC, A-BB, MM-A, UH, and J-CS contributed in review and editing. UH and J-CS contributed in resources, supervision, and funding acquisition.

### Conflict of Interest Statement

The authors declare that the research was conducted in the absence of any commercial or financial relationships that could be construed as a potential conflict of interest.

## Footnotes

^1^https://ecdc.europa.eu/

^2^https://ecdc.europa.eu
